# Supplementary material for: Validity of a Magnet-Based Timing System Using the Magnetometer Built into an IMU
Source: Sensors (Basel). 2021 Aug 27;21(17):5773. doi: 10.3390/s21175773 (PMC8433996; doi:10.3390/s21175773)
Supplement: Supplementary file 1 [file sensors-21-05773-s001.zip › sensors-1329185-supplementary.pdf]

**Running Context Photocell vs M-BTS**

| SECTIONS | SUBJECT | SECTION | DIST (m) | Photocell Time (s) | M-BTS Time (s) | X_Time | SD_Time | CV | Dif | SPEED (km/h) |
|---|---|---|---|---|---|---|---|---|---|---|
| Section 1 (0-5m) | SUBJECT 1 | 1 | 5 | 0,947 | 0,940 | 0,943 | 0,005 | 0,487 | 0,006 | 19,02 |
| | SUBJECT 2 | 1 | 5 | 0,888 | 0,820 | 0,854 | 0,048 | 5,630 | 0,068 | 20,27 |
| | SUBJECT 3 | 1 | 5 | 0,814 | 0,775 | 0,795 | 0,028 | 3,471 | 0,039 | 22,11 |
| | SUBJECT 4 | 1 | 5 | 0,819 | 0,765 | 0,792 | 0,038 | 4,778 | 0,054 | 21,99 |
| | SUBJECT 5 | 1 | 5 | 0,872 | 0,795 | 0,834 | 0,054 | 6,532 | 0,077 | 20,64 |
| | SUBJECT 6 | 1 | 5 | 0,787 | 0,775 | 0,781 | 0,008 | 1,042 | 0,012 | 22,89 |
| | SUBJECT 7 | 1 | 5 | 0,796 | 0,730 | 0,763 | 0,047 | 6,117 | 0,066 | 22,61 |
| | SUBJECT 8 | 1 | 5 | 0,832 | 0,780 | 0,806 | 0,036 | 4,520 | 0,051 | 21,65 |
| | SUBJECT 9 | 1 | 5 | 0,829 | 0,785 | 0,807 | 0,031 | 3,813 | 0,044 | 21,73 |
| | SUBJECT 10 | 1 | 5 | 0,755 | 0,730 | 0,743 | 0,018 | 2,381 | 0,025 | 23,84 |
| | SUBJECT 11 | 1 | 5 | 0,772 | 0,735 | 0,753 | 0,026 | 3,426 | 0,037 | 23,33 |
| | SUBJECT 12 | 1 | 5 | 0,765 | 0,750 | 0,758 | 0,011 | 1,400 | 0,015 | 23,53 |
| | SUBJECT 13 | 1 | 5 | 0,719 | 0,700 | 0,709 | 0,013 | 1,844 | 0,019 | 25,05 |
| | SUBJECT 14 | 1 | 5 | 0,745 | 0,705 | 0,725 | 0,028 | 3,854 | 0,040 | 24,18 |
| | SUBJECT 15 | 1 | 5 | 0,833 | 0,810 | 0,822 | 0,016 | 1,980 | 0,023 | 21,61 |
| | SUBJECT 16 | 1 | 5 | 0,775 | 0,740 | 0,757 | 0,024 | 3,222 | 0,035 | 23,24 |
| | SUBJECT 17 | 1 | 5 | 0,883 | 0,815 | 0,849 | 0,048 | 5,664 | 0,068 | 20,39 |
| | SUBJECT 18 | 1 | 5 | 0,745 | 0,730 | 0,737 | 0,010 | 1,391 | 0,015 | 24,18 |
| | SUBJECT 19 | 1 | 5 | 0,732 | 0,745 | 0,739 | 0,009 | 1,245 | -0,013 | 24,59 |
| | SUBJECT 20 | 1 | 5 | 0,725 | 0,725 | 0,725 | 0,000 | 0,000 | 0,000 | 24,83 |
| | SUBJECT 21 | 1 | 5 | 0,777 | 0,750 | 0,764 | 0,019 | 2,501 | 0,027 | 23,17 |
| | SUBJECT 22 | 1 | 5 | 0,823 | 0,795 | 0,809 | 0,020 | 2,447 | 0,028 | 21,87 |
| | SUBJECT 23 | 1 | 5 | 0,806 | 0,785 | 0,795 | 0,014 | 1,823 | 0,021 | 22,35 |
| | SUBJECT 24 | 1 | 5 | 0,732 | 0,735 | 0,733 | 0,002 | 0,338 | -0,003 | 24,61 |
| | SUBJECT 25 | 1 | 5 | 0,832 | 0,830 | 0,831 | 0,001 | 0,170 | 0,002 | 21,63 |
| | SUBJECT 26 | 1 | 5 | 0,913 | 0,870 | 0,891 | 0,030 | 3,372 | 0,043 | 19,73 |
| | SUBJECT 27 | 1 | 5 | 0,735 | 0,720 | 0,727 | 0,010 | 1,410 | 0,015 | 24,51 |
| | SUBJECT 28 | 1 | 5 | 0,832 | 0,805 | 0,818 | 0,019 | 2,290 | 0,027 | 21,65 |
| | SUBJECT 29 | 1 | 5 | 0,731 | 0,700 | 0,715 | 0,022 | 3,015 | 0,031 | 24,64 |
| | SUBJECT 30 | 1 | 5 | 0,727 | 0,715 | 0,721 | 0,008 | 1,128 | 0,012 | 24,78 |
| | SUBJECT 31 | 1 | 5 | 0,704 | 0,670 | 0,687 | 0,024 | 3,449 | 0,034 | 25,59 |
| | SUBJECT 32 | 1 | 5 | 0,741 | 0,685 | 0,713 | 0,039 | 5,506 | 0,055 | 24,31 |
| | SUBJECT 33 | 1 | 5 | 0,709 | 0,715 | 0,712 | 0,005 | 0,646 | -0,007 | 25,41 |
| | SUBJECT 34 | 1 | 5 | 0,823 | 0,755 | 0,789 | 0,048 | 6,051 | 0,068 | 21,88 |
| | SUBJECT 35 | 1 | 5 | 0,802 | 0,745 | 0,773 | 0,040 | 5,167 | 0,057 | 22,46 |
| | SUBJECT 36 | 1 | 5 | 0,786 | 0,735 | 0,760 | 0,036 | 4,697 | 0,051 | 22,92 |
| | SUBJECT 37 | 1 | 5 | 0,748 | 0,755 | 0,751 | 0,005 | 0,706 | -0,007 | 24,08 |
| | SUBJECT 38 | 1 | 5 | 0,804 | 0,760 | 0,782 | 0,031 | 3,979 | 0,044 | 22,39 |
| | SUBJECT 39 | 1 | 5 | 0,771 | 0,725 | 0,748 | 0,032 | 4,303 | 0,046 | 23,36 |
| | SUBJECT 40 | 1 | 5 | 0,778 | 0,730 | 0,754 | 0,034 | 4,456 | 0,048 | 23,15 |
| | SUBJECT 41 | 1 | 5 | 0,790 | 0,735 | 0,762 | 0,039 | 5,056 | 0,055 | 22,80 |
| | SUBJECT 42 | 1 | 5 | 0,699 | 0,670 | 0,685 | 0,021 | 2,996 | 0,029 | 25,75 |
| | SUBJECT 43 | 1 | 5 | 0,857 | 0,825 | 0,841 | 0,022 | 2,649 | 0,032 | 21,02 |
| Section 2 (5-10m) | SUBJECT 1 | 2 | 5 | 0,891 | 0,845 | 0,868 | 0,033 | 3,747 | 0,046 | 20,20 |
| | SUBJECT 2 | 2 | 5 | 0,710 | 0,705 | 0,707 | 0,003 | 0,450 | 0,005 | 25,37 |
| | SUBJECT 3 | 2 | 5 | 0,674 | 0,645 | 0,660 | 0,021 | 3,109 | 0,029 | 26,71 |
| | SUBJECT 4 | 2 | 5 | 0,686 | 0,670 | 0,678 | 0,011 | 1,669 | 0,016 | 26,24 |
| | SUBJECT 5 | 2 | 5 | 0,692 | 0,685 | 0,689 | 0,005 | 0,719 | 0,007 | 26,01 |
| | SUBJECT 6 | 2 | 5 | 0,718 | 0,685 | 0,701 | 0,023 | 3,277 | 0,033 | 25,09 |
| | SUBJECT 7 | 2 | 5 | 0,666 | 0,650 | 0,658 | 0,011 | 1,719 | 0,016 | 27,03 |
| | SUBJECT 8 | 2 | 5 | 0,693 | 0,675 | 0,684 | 0,013 | 1,861 | 0,018 | 25,97 |
| | SUBJECT 9 | 2 | 5 | 0,706 | 0,690 | 0,698 | 0,011 | 1,571 | 0,016 | 25,51 |
| | SUBJECT 10 | 2 | 5 | 0,678 | 0,655 | 0,666 | 0,016 | 2,388 | 0,023 | 26,57 |
| | SUBJECT 11 | 2 | 5 | 0,648 | 0,650 | 0,649 | 0,002 | 0,272 | -0,003 | 27,80 |
| | SUBJECT 12 | 2 | 5 | 0,721 | 0,650 | 0,685 | 0,050 | 7,275 | 0,070 | 24,98 |
| | SUBJECT 13 | 2 | 5 | 0,649 | 0,640 | 0,644 | 0,006 | 0,933 | 0,009 | 27,76 |
| | SUBJECT 14 | 2 | 5 | 0,650 | 0,645 | 0,648 | 0,004 | 0,546 | 0,005 | 27,69 |
| | SUBJECT 15 | 2 | 5 | 0,720 | 0,745 | 0,733 | 0,018 | 2,413 | -0,025 | 25,00 |
| | SUBJECT 16 | 2 | 5 | 0,682 | 0,680 | 0,681 | 0,001 | 0,156 | 0,001 | 26,41 |
| | SUBJECT 17 | 2 | 5 | 0,768 | 0,745 | 0,757 | 0,016 | 2,150 | 0,023 | 23,44 |
| | SUBJECT 18 | 2 | 5 | 0,650 | 0,635 | 0,642 | 0,010 | 1,596 | 0,015 | 27,71 |
| | SUBJECT 19 | 2 | 5 | 0,659 | 0,665 | 0,662 | 0,004 | 0,641 | -0,006 | 27,31 |
| | SUBJECT 20 | 2 | 5 | 0,680 | 0,660 | 0,670 | 0,014 | 2,059 | 0,020 | 26,49 |
| | SUBJECT 21 | 2 | 5 | 0,690 | 0,685 | 0,687 | 0,003 | 0,463 | 0,004 | 26,11 |
| | SUBJECT 22 | 2 | 5 | 0,707 | 0,715 | 0,711 | 0,006 | 0,796 | -0,008 | 25,46 |
| | SUBJECT 23 | 2 | 5 | 0,700 | 0,695 | 0,698 | 0,004 | 0,507 | 0,005 | 25,71 |
| | SUBJECT 24 | 2 | 5 | 0,694 | 0,655 | 0,675 | 0,028 | 4,089 | 0,039 | 25,94 |
| | SUBJECT 25 | 2 | 5 | 0,756 | 0,735 | 0,745 | 0,014 | 1,945 | 0,021 | 23,83 |
| | SUBJECT 26 | 2 | 5 | 0,806 | 0,785 | 0,796 | 0,015 | 1,867 | 0,021 | 22,33 |
| | SUBJECT 27 | 2 | 5 | 0,647 | 0,640 | 0,644 | 0,005 | 0,769 | 0,007 | 27,82 |
| | SUBJECT 28 | 2 | 5 | 0,754 | 0,715 | 0,734 | 0,027 | 3,708 | 0,039 | 23,89 |
| | SUBJECT 29 | 2 | 5 | 0,649 | 0,655 | 0,652 | 0,004 | 0,651 | -0,006 | 27,73 |
| | SUBJECT 30 | 2 | 5 | 0,666 | 0,655 | 0,660 | 0,007 | 1,125 | 0,011 | 27,05 |
| | SUBJECT 31 | 2 | 5 | 0,653 | 0,615 | 0,634 | 0,027 | 4,238 | 0,038 | 27,57 |
| | SUBJECT 32 | 2 | 5 | 0,608 | 0,605 | 0,606 | 0,002 | 0,292 | 0,002 | 29,63 |
| | SUBJECT 33 | 2 | 5 | 0,650 | 0,615 | 0,633 | 0,025 | 3,913 | 0,035 | 27,69 |
| | SUBJECT 34 | 2 | 5 | 0,651 | 0,670 | 0,661 | 0,013 | 2,034 | -0,019 | 27,65 |
| | SUBJECT 35 | 2 | 5 | 0,684 | 0,660 | 0,672 | 0,017 | 2,525 | 0,024 | 26,32 |
| | SUBJECT 36 | 2 | 5 | 0,671 | 0,660 | 0,666 | 0,008 | 1,169 | 0,011 | 26,83 |
| | SUBJECT 37 | 2 | 5 | 0,697 | 0,680 | 0,688 | 0,012 | 1,695 | 0,017 | 25,84 |
| | SUBJECT 38 | 2 | 5 | 0,729 | 0,705 | 0,717 | 0,017 | 2,367 | 0,024 | 24,69 |
| | SUBJECT 39 | 2 | 5 | 0,669 | 0,650 | 0,659 | 0,013 | 1,984 | 0,019 | 26,93 |
| | SUBJECT 40 | 2 | 5 | 0,682 | 0,660 | 0,671 | 0,015 | 2,267 | 0,022 | 26,41 |
| | SUBJECT 41 | 2 | 5 | 0,671 | 0,670 | 0,671 | 0,001 | 0,105 | 0,001 | 26,83 |
| | SUBJECT 42 | 2 | 5 | 0,652 | 0,615 | 0,633 | 0,026 | 4,076 | 0,037 | 27,63 |
| | SUBJECT 43 | 2 | 5 | 0,770 | 0,750 | 0,760 | 0,014 | 1,815 | 0,020 | 23,39 |
| Section 3 (10-20m) | SUBJECT 1 | 3 | 10 | 1,508 | 1,510 | 1,509 | 0,002 | 0,117 | -0,002 | 23,88 |
| | SUBJECT 2 | 3 | 10 | 1,301 | 1,295 | 1,298 | 0,004 | 0,327 | 0,006 | 27,67 |
| | SUBJECT 3 | 3 | 10 | 1,235 | 1,220 | 1,227 | 0,010 | 0,835 | 0,015 | 29,16 |
| | SUBJECT 4 | 3 | 10 | 1,268 | 1,260 | 1,264 | 0,006 | 0,448 | 0,008 | 28,39 |
| | SUBJECT 5 | 3 | 10 | 1,290 | 1,275 | 1,282 | 0,010 | 0,800 | 0,015 | 27,92 |
| | SUBJECT 6 | 3 | 10 | 1,321 | 1,310 | 1,315 | 0,007 | 0,565 | 0,011 | 27,26 |
| | SUBJECT 7 | 3 | 10 | 1,232 | 1,240 | 1,236 | 0,006 | 0,458 | -0,008 | 29,22 |
| | SUBJECT 8 | 3 | 10 | 1,268 | 1,275 | 1,271 | 0,005 | 0,417 | -0,007 | 28,40 |
| | SUBJECT 9 | 3 | 10 | 1,337 | 1,340 | 1,338 | 0,002 | 0,185 | -0,004 | 26,94 |
| | SUBJECT 10 | 3 | 10 | 1,254 | 1,235 | 1,244 | 0,013 | 1,051 | 0,019 | 28,72 |
| | SUBJECT 11 | 3 | 10 | 1,272 | 1,270 | 1,271 | 0,001 | 0,111 | 0,002 | 28,30 |
| | SUBJECT 12 | 3 | 10 | 1,242 | 1,275 | 1,258 | 0,024 | 1,883 | -0,034 | 29,00 |
| | SUBJECT 13 | 3 | 10 | 1,220 | 1,205 | 1,213 | 0,011 | 0,875 | 0,015 | 29,51 |
| | SUBJECT 14 | 3 | 10 | 1,256 | 1,240 | 1,248 | 0,011 | 0,878 | 0,016 | 28,67 |
| | SUBJECT 15 | 3 | 10 | 1,480 | 1,455 | 1,467 | 0,017 | 1,181 | 0,025 | 24,33 |
| | SUBJECT 16 | 3 | 10 | 1,351 | 1,335 | 1,343 | 0,011 | 0,842 | 0,016 | 26,65 |
| | SUBJECT 17 | 3 | 10 | 1,422 | 1,430 | 1,426 | 0,006 | 0,397 | -0,008 | 25,32 |
| | SUBJECT 18 | 3 | 10 | 1,228 | 1,200 | 1,214 | 0,020 | 1,631 | 0,028 | 29,32 |
| | SUBJECT 19 | 3 | 10 | 1,271 | 1,230 | 1,250 | 0,029 | 2,291 | 0,041 | 28,34 |
| | SUBJECT 20 | 3 | 10 | 1,241 | 1,235 | 1,238 | 0,004 | 0,314 | 0,006 | 29,02 |
| | SUBJECT 21 | 3 | 10 | 1,275 | 1,275 | 1,275 | 0,000 | 0,028 | 0,000 | 28,25 |
| | SUBJECT 22 | 3 | 10 | 1,312 | 1,320 | 1,316 | 0,006 | 0,430 | -0,008 | 27,44 |
| | SUBJECT 23 | 3 | 10 | 1,301 | 1,290 | 1,296 | 0,008 | 0,600 | 0,011 | 27,67 |
| | SUBJECT 24 | 3 | 10 | 1,240 | 1,240 | 1,240 | 0,000 | 0,000 | 0,000 | 29,03 |
| | SUBJECT 25 | 3 | 10 | 1,414 | 1,415 | 1,415 | 0,001 | 0,050 | -0,001 | 25,46 |
| | SUBJECT 26 | 3 | 10 | 1,529 | 1,535 | 1,532 | 0,005 | 0,300 | -0,007 | 23,55 |
| | SUBJECT 27 | 3 | 10 | 1,267 | 1,250 | 1,258 | 0,012 | 0,927 | 0,017 | 28,42 |
| | SUBJECT 28 | 3 | 10 | 1,419 | 1,405 | 1,412 | 0,010 | 0,676 | 0,014 | 25,38 |
| | SUBJECT 29 | 3 | 10 | 1,294 | 1,285 | 1,290 | 0,006 | 0,494 | 0,009 | 27,82 |
| | SUBJECT 30 | 3 | 10 | 1,279 | 1,280 | 1,279 | 0,001 | 0,083 | -0,001 | 28,16 |
| | SUBJECT 31 | 3 | 10 | 1,136 | 1,145 | 1,141 | 0,006 | 0,558 | -0,009 | 31,69 |
| | SUBJECT 32 | 3 | 10 | 1,151 | 1,140 | 1,146 | 0,008 | 0,679 | 0,011 | 31,28 |
| | SUBJECT 33 | 3 | 10 | 1,217 | 1,195 | 1,206 | 0,016 | 1,290 | 0,022 | 29,58 |
| | SUBJECT 34 | 3 | 10 | 1,323 | 1,270 | 1,297 | 0,037 | 2,891 | 0,053 | 27,21 |
| | SUBJECT 35 | 3 | 10 | 1,279 | 1,260 | 1,269 | 0,013 | 1,031 | 0,019 | 28,16 |
| | SUBJECT 36 | 3 | 10 | 1,256 | 1,255 | 1,256 | 0,001 | 0,056 | 0,001 | 28,66 |
| | SUBJECT 37 | 3 | 10 | 1,313 | 1,285 | 1,299 | 0,020 | 1,524 | 0,028 | 27,42 |
| | SUBJECT 38 | 3 | 10 | 1,387 | 1,375 | 1,381 | 0,008 | 0,614 | 0,012 | 25,96 |
| | SUBJECT 39 | 3 | 10 | 1,234 | 1,220 | 1,227 | 0,010 | 0,807 | 0,014 | 29,17 |
| | SUBJECT 40 | 3 | 10 | 1,231 | 1,230 | 1,231 | 0,001 | 0,057 | 0,001 | 29,24 |
| | SUBJECT 41 | 3 | 10 | 1,290 | 1,285 | 1,288 | 0,004 | 0,275 | 0,005 | 27,91 |
| | SUBJECT 42 | 3 | 10 | 1,180 | 1,165 | 1,172 | 0,010 | 0,875 | 0,015 | 30,52 |
| | SUBJECT 43 | 3 | 10 | 1,444 | 1,440 | 1,442 | 0,002 | 0,172 | 0,004 | 24,94 |
| Section 4 (20-40m) | SUBJECT 1 | 4 | 20 | 2,980 | 2,980 | 2,980 | 0,000 | 0,012 | -0,001 | 24,17 |
| | SUBJECT 2 | 4 | 20 | 2,516 | 2,510 | 2,513 | 0,004 | 0,169 | 0,006 | 28,62 |
| | SUBJECT 3 | 4 | 20 | 2,290 | 2,285 | 2,287 | 0,003 | 0,139 | 0,004 | 31,45 |
| | SUBJECT 4 | 4 | 20 | 2,417 | 2,415 | 2,416 | 0,001 | 0,059 | 0,002 | 29,79 |
| | SUBJECT 5 | 4 | 20 | 2,452 | 2,450 | 2,451 | 0,001 | 0,058 | 0,002 | 29,36 |
| | SUBJECT 6 | 4 | 20 | 2,529 | 2,545 | 2,537 | 0,012 | 0,460 | -0,016 | 28,48 |
| | SUBJECT 7 | 4 | 20 | 2,352 | 2,350 | 2,351 | 0,001 | 0,060 | 0,002 | 30,61 |
| | SUBJECT 8 | 4 | 20 | 2,466 | 2,465 | 2,465 | 0,000 | 0,014 | 0,001 | 29,20 |
| | SUBJECT 9 | 4 | 20 | 2,647 | 2,640 | 2,644 | 0,005 | 0,187 | 0,007 | 27,20 |
| | SUBJECT 10 | 4 | 20 | 2,495 | 2,475 | 2,485 | 0,014 | 0,569 | 0,020 | 28,86 |
| | SUBJECT 11 | 4 | 20 | 2,452 | 2,445 | 2,449 | 0,005 | 0,202 | 0,007 | 29,36 |
| | SUBJECT 12 | 4 | 20 | 2,449 | 2,455 | 2,452 | 0,005 | 0,187 | -0,006 | 29,41 |
| | SUBJECT 13 | 4 | 20 | 2,416 | 2,415 | 2,416 | 0,001 | 0,029 | 0,001 | 29,80 |
| | SUBJECT 14 | 4 | 20 | 2,430 | 2,430 | 2,430 | 0,000 | 0,000 | 0,000 | 29,63 |
| | SUBJECT 15 | 4 | 20 | 2,958 | 2,955 | 2,957 | 0,002 | 0,072 | 0,003 | 24,34 |
| | SUBJECT 16 | 4 | 20 | 2,657 | 2,670 | 2,664 | 0,009 | 0,345 | -0,013 | 27,10 |
| | SUBJECT 17 | 4 | 20 | 2,854 | 2,855 | 2,855 | 0,001 | 0,025 | -0,001 | 25,23 |
| | SUBJECT 18 | 4 | 20 | 2,324 | 2,310 | 2,317 | 0,010 | 0,412 | 0,014 | 30,99 |
| | SUBJECT 19 | 4 | 20 | 2,419 | 2,415 | 2,417 | 0,002 | 0,102 | 0,003 | 29,77 |
| | SUBJECT 20 | 4 | 20 | 2,440 | 2,425 | 2,432 | 0,010 | 0,422 | 0,015 | 29,51 |
| | SUBJECT 21 | 4 | 20 | 2,544 | 2,530 | 2,537 | 0,010 | 0,390 | 0,014 | 28,30 |
| | SUBJECT 22 | 4 | 20 | 2,555 | 2,515 | 2,535 | 0,028 | 1,102 | 0,039 | 28,19 |
| | SUBJECT 23 | 4 | 20 | 2,512 | 2,500 | 2,506 | 0,008 | 0,339 | 0,012 | 28,66 |
| | SUBJECT 24 | 4 | 20 | 2,401 | 2,400 | 2,401 | 0,001 | 0,029 | 0,001 | 29,99 |
| | SUBJECT 25 | 4 | 20 | 2,832 | 2,825 | 2,828 | 0,005 | 0,163 | 0,006 | 25,43 |
| | SUBJECT 26 | 4 | 20 | 3,134 | 3,135 | 3,134 | 0,001 | 0,034 | -0,002 | 22,98 |
| | SUBJECT 27 | 4 | 20 | 2,448 | 2,445 | 2,446 | 0,002 | 0,072 | 0,002 | 29,42 |
| | SUBJECT 28 | 4 | 20 | 2,779 | 2,800 | 2,790 | 0,015 | 0,532 | -0,021 | 25,91 |
| | SUBJECT 29 | 4 | 20 | 2,460 | 2,465 | 2,463 | 0,004 | 0,144 | -0,005 | 29,27 |
| | SUBJECT 30 | 4 | 20 | 2,491 | 2,510 | 2,501 | 0,013 | 0,537 | -0,019 | 28,90 |
| | SUBJECT 31 | 4 | 20 | 2,229 | 2,220 | 2,225 | 0,006 | 0,286 | 0,009 | 32,30 |
| | SUBJECT 32 | 4 | 20 | 2,245 | 2,255 | 2,250 | 0,007 | 0,314 | -0,010 | 32,07 |
| | SUBJECT 33 | 4 | 20 | 2,367 | 2,390 | 2,379 | 0,016 | 0,684 | -0,023 | 30,42 |
| | SUBJECT 34 | 4 | 20 | 2,524 | 2,525 | 2,525 | 0,001 | 0,028 | -0,001 | 28,53 |
| | SUBJECT 35 | 4 | 20 | 2,457 | 2,475 | 2,466 | 0,013 | 0,516 | -0,018 | 29,30 |
| | SUBJECT 36 | 4 | 20 | 2,437 | 2,450 | 2,444 | 0,009 | 0,376 | -0,013 | 29,54 |
| | SUBJECT 37 | 4 | 20 | 2,527 | 2,545 | 2,536 | 0,013 | 0,502 | -0,018 | 28,49 |
| | SUBJECT 38 | 4 | 20 | 2,755 | 2,770 | 2,763 | 0,011 | 0,384 | -0,015 | 26,13 |
| | SUBJECT 39 | 4 | 20 | 2,397 | 2,410 | 2,403 | 0,010 | 0,397 | -0,014 | 30,04 |
| | SUBJECT 40 | 4 | 20 | 2,474 | 2,460 | 2,467 | 0,010 | 0,401 | 0,014 | 29,10 |
| | SUBJECT 41 | 4 | 20 | 2,481 | 2,475 | 2,478 | 0,004 | 0,157 | 0,006 | 29,03 |
| | SUBJECT 42 | 4 | 20 | 2,263 | 2,290 | 2,277 | 0,019 | 0,839 | -0,027 | 31,82 |
| | SUBJECT 43 | 4 | 20 | 2,953 | 2,935 | 2,944 | 0,013 | 0,432 | 0,018 | 24,38 |
| Section 5 (40-60m) | SUBJECT 1 | 5 | 20 | 3,107 | 3,105 | 3,106 | 0,001 | 0,046 | 0,002 | 23,17 |
| | SUBJECT 2 | 5 | 20 | 2,566 | 2,555 | 2,561 | 0,008 | 0,304 | 0,011 | 28,06 |
| | SUBJECT 3 | 5 | 20 | 2,289 | 2,290 | 2,289 | 0,001 | 0,046 | -0,002 | 31,46 |
| | SUBJECT 4 | 5 | 20 | 2,461 | 2,465 | 2,463 | 0,003 | 0,115 | -0,004 | 29,26 |
| | SUBJECT 5 | 5 | 20 | 2,532 | 2,550 | 2,541 | 0,013 | 0,501 | -0,018 | 28,44 |
| | SUBJECT 6 | 5 | 20 | 2,521 | 2,535 | 2,528 | 0,010 | 0,392 | -0,014 | 28,56 |
| | SUBJECT 7 | 5 | 20 | 2,413 | 2,425 | 2,419 | 0,009 | 0,365 | -0,013 | 29,84 |
| | SUBJECT 8 | 5 | 20 | 2,555 | 2,545 | 2,550 | 0,007 | 0,277 | 0,010 | 28,18 |
| | SUBJECT 9 | 5 | 20 | 2,779 | 2,800 | 2,790 | 0,015 | 0,532 | -0,021 | 25,91 |
| | SUBJECT 10 | 5 | 20 | 2,549 | 2,555 | 2,552 | 0,005 | 0,180 | -0,006 | 28,25 |
| | SUBJECT 11 | 5 | 20 | 2,425 | 2,440 | 2,433 | 0,011 | 0,436 | -0,015 | 29,69 |
| | SUBJECT 12 | 5 | 20 | 2,581 | 2,545 | 2,563 | 0,025 | 0,993 | 0,036 | 27,90 |
| | SUBJECT 13 | 5 | 20 | 2,480 | 2,485 | 2,483 | 0,004 | 0,142 | -0,005 | 29,03 |
| | SUBJECT 14 | 5 | 20 | 2,451 | 2,465 | 2,458 | 0,010 | 0,403 | -0,014 | 29,38 |
| | SUBJECT 15 | 5 | 20 | 3,083 | 3,080 | 3,081 | 0,002 | 0,057 | 0,002 | 23,36 |
| | SUBJECT 16 | 5 | 20 | 2,775 | 2,780 | 2,778 | 0,004 | 0,127 | -0,005 | 25,95 |
| | SUBJECT 17 | 5 | 20 | 3,022 | 3,020 | 3,021 | 0,001 | 0,035 | 0,002 | 23,83 |
| | SUBJECT 18 | 5 | 20 | 2,362 | 2,355 | 2,358 | 0,005 | 0,195 | 0,006 | 30,49 |
| | SUBJECT 19 | 5 | 20 | 2,525 | 2,515 | 2,520 | 0,007 | 0,267 | 0,009 | 28,52 |
| | SUBJECT 20 | 5 | 20 | 2,517 | 2,530 | 2,524 | 0,009 | 0,364 | -0,013 | 28,61 |
| | SUBJECT 21 | 5 | 20 | 2,600 | 2,605 | 2,603 | 0,004 | 0,136 | -0,005 | 27,69 |
| | SUBJECT 22 | 5 | 20 | 2,566 | 2,590 | 2,578 | 0,017 | 0,672 | -0,024 | 28,06 |
| | SUBJECT 23 | 5 | 20 | 2,552 | 2,540 | 2,546 | 0,008 | 0,333 | 0,012 | 28,21 |
| | SUBJECT 24 | 5 | 20 | 2,439 | 2,445 | 2,442 | 0,005 | 0,188 | -0,007 | 29,53 |
| | SUBJECT 25 | 5 | 20 | 2,933 | 2,950 | 2,941 | 0,012 | 0,421 | -0,018 | 24,55 |
| | SUBJECT 26 | 5 | 20 | 3,223 | 3,230 | 3,226 | 0,005 | 0,164 | -0,007 | 22,34 |
| | SUBJECT 27 | 5 | 20 | 2,660 | 2,675 | 2,668 | 0,011 | 0,398 | -0,015 | 27,07 |
| | SUBJECT 28 | 5 | 20 | 2,879 | 2,885 | 2,882 | 0,004 | 0,147 | -0,006 | 25,01 |
| | SUBJECT 29 | 5 | 20 | 2,452 | 2,445 | 2,449 | 0,005 | 0,202 | 0,007 | 29,36 |
| | SUBJECT 30 | 5 | 20 | 2,639 | 2,635 | 2,637 | 0,002 | 0,094 | 0,004 | 27,29 |
| | SUBJECT 31 | 5 | 20 | 2,239 | 2,255 | 2,247 | 0,011 | 0,504 | -0,016 | 32,16 |
| | SUBJECT 32 | 5 | 20 | 2,337 | 2,345 | 2,341 | 0,006 | 0,242 | -0,008 | 30,81 |
| | SUBJECT 33 | 5 | 20 | 2,404 | 2,405 | 2,404 | 0,001 | 0,044 | -0,002 | 29,96 |
| | SUBJECT 34 | 5 | 20 | 2,593 | 2,620 | 2,606 | 0,019 | 0,746 | -0,027 | 27,77 |
| | SUBJECT 35 | 5 | 20 | 2,581 | 2,575 | 2,578 | 0,004 | 0,165 | 0,006 | 27,90 |
| | SUBJECT 36 | 5 | 20 | 2,535 | 2,525 | 2,530 | 0,007 | 0,279 | 0,010 | 28,40 |
| | SUBJECT 37 | 5 | 20 | 2,671 | 2,640 | 2,655 | 0,022 | 0,812 | 0,031 | 26,96 |
| | SUBJECT 38 | 5 | 20 | 2,851 | 2,870 | 2,861 | 0,013 | 0,470 | -0,019 | 25,25 |
| | SUBJECT 39 | 5 | 20 | 2,415 | 2,400 | 2,407 | 0,010 | 0,426 | 0,015 | 29,82 |
| | SUBJECT 40 | 5 | 20 | 2,584 | 2,595 | 2,590 | 0,008 | 0,300 | -0,011 | 27,86 |
| | SUBJECT 41 | 5 | 20 | 2,547 | 2,545 | 2,546 | 0,001 | 0,056 | 0,002 | 28,27 |
| | SUBJECT 42 | 5 | 20 | 2,369 | 2,360 | 2,364 | 0,006 | 0,254 | 0,009 | 30,40 |
| | SUBJECT 43 | 5 | 20 | 2,936 | 2,930 | 2,933 | 0,004 | 0,145 | 0,006 | 24,52 |

| | | Photocell Time (s) | M-BTS Time (s) | X_Time | SD_Time | CV | Dif | SPEED (km/h) |
|---|---|---|---|---|---|---|---|---|
| Section 1 (0-5m) | MEAN | 0,789 | 0,757 | 0,773 | 0,024 | 3,045 | 0,032 | 22,92 |
| | SD | 0,058 | 0,053 | 0,054 | 0,015 | 1,829 | 0,023 | 1,64 |
| Section 2 (5-10m) | MEAN | 0,693 | 0,677 | 0,685 | 0,013 | 1,929 | 0,016 | 26,11 |
| | SD | 0,050 | 0,047 | 0,048 | 0,010 | 1,449 | 0,018 | 1,72 |
| Section 3 (10-20m) | MEAN | 1,297 | 1,288 | 1,292 | 0,009 | 0,698 | 0,008 | 27,88 |
| | SD | 0,088 | 0,090 | 0,089 | 0,008 | 0,621 | 0,015 | 1,81 |
| Section 4 (20-40m) | MEAN | 2,530 | 2,531 | 2,530 | 0,007 | 0,283 | 0,000 | 28,63 |
| | SD | 0,208 | 0,207 | 0,208 | 0,006 | 0,246 | 0,013 | 2,18 |
| Section 5 (40-60m) | MEAN | 2,604 | 2,607 | 2,606 | 0,008 | 0,302 | -0,003 | 27,84 |
| | SD | 0,228 | 0,229 | 0,228 | 0,006 | 0,219 | 0,013 | 2,28 |

**Skiing Context Photocell vs M-BTS**

| SECTIONS | SUBJECT | SECTION | DIST (m) | Photocell Time (s) | M-BTS Time (s) | X_Time | SD_Time | CV | Dif | SPEED (km/h) |
|---|---|---|---|---|---|---|---|---|---|---|
| Section 1 (G1-2) | SUBJECT 1 | 1 | 10,5 | 1,165 | 1,230 | 1,198 | 0,046 | 3,838 | -0,065 | 32,45 |
| | SUBJECT 2 | 1 | 10,5 | 0,988 | 1,050 | 1,019 | 0,044 | 4,302 | -0,062 | 38,26 |
| | SUBJECT 3 | 1 | 10,5 | 1,090 | 1,060 | 1,075 | 0,021 | 1,973 | 0,030 | 34,68 |
| | SUBJECT 4 | 1 | 10,5 | 1,217 | 1,200 | 1,209 | 0,012 | 0,995 | 0,017 | 31,06 |
| | SUBJECT 5 | 1 | 10,5 | 1,169 | 1,200 | 1,185 | 0,022 | 1,851 | -0,031 | 32,34 |
| | SUBJECT 6 | 1 | 10,5 | 1,005 | 1,060 | 1,033 | 0,039 | 3,767 | -0,055 | 37,61 |
| | SUBJECT 7 | 1 | 10,5 | 1,141 | 1,080 | 1,111 | 0,043 | 3,884 | 0,061 | 33,13 |
| | SUBJECT 8 | 1 | 10,5 | 1,291 | 1,270 | 1,281 | 0,015 | 1,160 | 0,021 | 29,28 |
| Section 2 (G2-3) | SUBJECT 1 | 2 | 10,5 | 1,065 | 1,020 | 1,043 | 0,032 | 3,052 | 0,045 | 35,49 |
| | SUBJECT 2 | 2 | 10,5 | 1,028 | 0,970 | 0,999 | 0,041 | 4,105 | 0,058 | 36,77 |
| | SUBJECT 3 | 2 | 10,5 | 0,851 | 0,930 | 0,891 | 0,056 | 6,273 | -0,079 | 44,42 |
| | SUBJECT 4 | 2 | 10,5 | 0,986 | 0,990 | 0,988 | 0,003 | 0,286 | -0,004 | 38,34 |
| | SUBJECT 5 | 2 | 10,5 | 1,056 | 1,020 | 1,038 | 0,025 | 2,452 | 0,036 | 35,80 |
| | SUBJECT 6 | 2 | 10,5 | 1,005 | 0,950 | 0,978 | 0,039 | 3,979 | 0,055 | 37,61 |
| | SUBJECT 7 | 2 | 10,5 | 0,942 | 0,960 | 0,951 | 0,013 | 1,338 | -0,018 | 40,13 |
| | SUBJECT 8 | 2 | 10,5 | 1,060 | 1,020 | 1,040 | 0,028 | 2,720 | 0,040 | 35,66 |
| Section 3 (G3-4) | SUBJECT 1 | 3 | 10,5 | 1,234 | 1,240 | 1,237 | 0,004 | 0,343 | -0,006 | 30,63 |
| | SUBJECT 2 | 3 | 10,5 | 1,039 | 1,070 | 1,055 | 0,022 | 2,079 | -0,031 | 36,38 |
| | SUBJECT 3 | 3 | 10,5 | 1,154 | 1,120 | 1,137 | 0,024 | 2,114 | 0,034 | 32,76 |
| | SUBJECT 4 | 3 | 10,5 | 1,107 | 1,150 | 1,129 | 0,030 | 2,694 | -0,043 | 34,15 |
| | SUBJECT 5 | 3 | 10,5 | 1,107 | 1,110 | 1,109 | 0,002 | 0,191 | -0,003 | 34,15 |
| | SUBJECT 6 | 3 | 10,5 | 1,044 | 1,070 | 1,057 | 0,018 | 1,739 | -0,026 | 36,21 |
| | SUBJECT 7 | 3 | 10,5 | 1,062 | 1,090 | 1,076 | 0,020 | 1,840 | -0,028 | 35,59 |
| | SUBJECT 8 | 3 | 10,5 | 1,112 | 1,160 | 1,136 | 0,034 | 2,988 | -0,048 | 33,99 |

| | | Photocell Time (s) | M-BTS Time (s) | X_Time | SD_Time | CV | Dif | SPEED (km/h) |
|---|---|---|---|---|---|---|---|---|
| Section 1 (G1-2) | MEAN | 1,133 | 1,144 | 1,139 | 0,030 | 2,721 | -0,011 | 33,60 |
| | SD | 0,103 | 0,090 | 0,093 | 0,014 | 1,359 | 0,049 | 3,10 |
| Section 2 (G2-3) | MEAN | 0,999 | 0,983 | 0,991 | 0,030 | 3,026 | 0,017 | 38,03 |
| | SD | 0,073 | 0,035 | 0,052 | 0,017 | 1,828 | 0,047 | 3,03 |
| Section 3 (G3-4) | MEAN | 1,107 | 1,126 | 1,117 | 0,019 | 1,749 | -0,019 | 34,23 |
| | SD | 0,064 | 0,057 | 0,059 | 0,011 | 1,006 | 0,027 | 1,91 |
